# Organobentonites Modified with Poly(Acrylic Acid) and Its Sodium Salt for Foundry Applications

**DOI:** 10.3390/ma14081947

**Published:** 2021-04-13

**Authors:** Sylwia Cukrowicz, Maciej Sitarz, Kamil Kornaus, Karolina Kaczmarska, Artur Bobrowski, Agnieszka Gubernat, Beata Grabowska

**Affiliations:** 1 Faculty of Foundry Engineering, AGH-University of Science and Technology, Reymonta 23, 30-059 Krakow, Poland; karolina.kaczmarska@agh.edu.pl (K.K.); arturb@agh.edu.pl (A.B.); beata.grabowska@agh.edu.pl (B.G.); 2 Faculty of Materials Science and Ceramics, AGH-University of Science and Technology, Mickiewicza 30, 30-059 Krakow, Poland; msitarz@agh.edu.pl (M.S.); kornaus@agh.edu.pl (K.K.); gubernat@agh.edu.pl (A.G.)

**Keywords:** bentonite, montmorillonite, poly(acrylic acid), composites, foundry, adsorption, intercalation

## Abstract

The article aims to verify the possibility of obtaining an organic–inorganic material acting as both a binder and a lustrous carbon carrier in bentonite-bonded molding sands. Due to the wide industrial application, organoclays can be considered as innovative materials supporting the foundry technology in meeting environmental requirements. In this study, the organic modification of montmorillonite in calcium bentonite (SN) was performed by poly(acrylic acid) (PAA) and its sodium salt (PAA/Na). Additionally, for the purpose of comparison, the sodium-activated bentonite/poly(acrylic acid) (SN-Na/PAA) composites were also prepared. The collective analysis of the research results used in the assessment of the mineral/polymer interaction mechanism indicates surface adsorption combined with the intercalation of PAA monolayer into the mineral interlayer spaces. Materials were characterized by the combination of Fourier-transform infrared spectroscopy (FTIR), X-ray diffraction (XRD), Brunauer–Emmett–Teller (BET) surface area analysis and scanning electron microscopy/energy dispersive spectroscopy (SEM/EDS) methods. Based on the XRD analysis, the influence of PAA/Na on the aluminosilicate layered structure was found to be destructive, which may adversely affect the binding properties of SN/PAA/Na composites considered as a potential group of new foundry binders. The SN/PAA and SN-Na/PPA composites (with appropriate polymer content) can act as a binding agent in the synthetic molding sand technology, despite coating the bentonite particles with polymer molecules. The risk of losing the mineral′s binding capacity is reduced by the good binding properties of pol(acrylic acid) itself. The article is the first stage (preceding the thermal analysis and the strength tests of molding sands with the prepared organobentonites) in determining the possibility of obtaining a new full-value foundry binder in molding sands with bentonite.

## 1. Introduction

Ongoing materials science and material engineering development call for the constant efficiency improvement of modification processes of well-known materials. Organic-inorganic composites/hybrids are promising alternative materials to those synthesized for research purpose only. Thanks to their unique properties and multi-functionality, they offer many application prospects in extremely diverse fields of industries [1,2]. Among the organic-inorganic complex materials, the most popular are the organically modified layered minerals, often referred to as organoclays [3]. The modification usually results in an interlayer ion exchange between hydrated inorganic and organic cations, causing an increase in the hydrophobicity of the clay. Therefore, such materials are used as fillers in polymer-clay nanocomposites or also as adsorbents of organic pollutants [4,5,6]. The interest in clay minerals results mainly from their availability in the form of numerous deposits and diversity in terms of structure and chemical composition, which combined with the knowledge of numerous methods of their modification, favor targeted modification [7]. The potential of organoclays is the driving force for the attempt to develop new binding material in synthetic molding sands (with bentonite) technology.

Synthetic molding sands are mixtures of mineral matrix, bentonite, water and carbon additives. Carbon carriers in the form of coal dust or synthetic resins are the essential ingredient preventing the occurrence of casting defects, e.g., burns [8]. On the other hand, they are a source of increased emissions of highly harmful gaseous compounds, such as volatile organic compounds (VOCs), polycyclic aromatic hydrocarbons (PAHs) and benzene, toluene, ethylbenzene and xylenes (BTEX). The products of their high-temperature pyrolysis released while pouring the mold with liquid metal get into the atmosphere and remain in the structure of the waste molding sands, being a threat during the storage or recycling of the circulating molding sand in the foundry [9,10,11]. Because of environment protection and improvement of work conditions, global leaders in supplying additives for bentonite-bonded sands are searching for commonly used carbon additive substitutes being effective, but at the same time more environmentally-friendly [12,13]. The research involves both independent materials with a sufficiently high lustrous carbon forming capacity and multi-component systems of a good quality coal dust or brown coal and the materials that intensify their action and reduce the amount of a harmful gas emissions.

The patent application US5688313A presents a foundry sand additive consisting of activated carbon and/or activated graphite together with components capable of their in situ forming during the molding process, such as humic acid (salt)-containing ore, e.g., leonardite (FloCarb). The in situ activated carbon and/or graphite adsorb gaseous volatile organic compounds (VOCs) within the mold, reducing its emissions [14]. FloCarb is an organic, clean-burning carbon carrier that, according to the manufacturer′s technical data, improves the properties of synthetic molding sands, such as a compression strength and shakeout, while reducing the amount of PAH, VOC and BTEX emissions to about 50%. The possibility of replacing coal dust with other forms of coal, e.g., graphite, anthracite, amorphous carbon or their mixture, was also investigated [15]. The reduction of emissions and the presence of BTX compounds in waste molding sands was recorded, while maintaining high quality of the casting surfaces. IMERYS Metalcasting Solutions offers an ecological alternative to the classic components of synthetic molding sands called ENVIBOND. It is a binder based on modification with graphite and zeolite sodium bentonite (not a mixture but a structurally changed form of the mineral). By changing the wetting angle, graphite reduces mold penetration and increases compaction. The zeolite replaces the carbon residues and improves the capability of the mold adsorption [16]. Thanks to this formula, VOC emissions can be reduced over 50% (depending on the individual casting practice) [17]. Another study investigates the suitability of various wood flours (sawmill industry waste) as a component of bentonite-bonded molding sand, which resulted in the improvement of some of its strength properties [18]. Despite the authors′ suggestions regarding the environment beneficial effects of such additives, no studies have been conducted in this area. The ready mixtures of high quality coal dust and other lustrous carbon carriers are also very popular [13,19]. According to the manufacturers′ assurances, these carbon additives constitute a solution supporting foundries in adjusting the technology to new environmental standards. Nevertheless, at high temperatures, there is a risk of harmful compound emissions from the saturated hydrocarbon, nitrile (RCN) or alkyne (CH_2n−2_) groups [19].

The literature analysis indicates that several successful investigations in the field of development of ecological substitutes for coal dust have been carried out. However, their share in the world production of iron castings remains small. In this study, the attempt to develop a new, less complex binder in the technology of synthetic molding sands in the form of organically modified bentonite, acting at the same time as a substitute for popular carbon additives, is an innovative approach to solving the problem of their harmfulness based on the high-temperature behavior of selected hydrophilic polymers. The additional motivation is the possibility of using the already known and extensive knowledge in the field of properties and applications of other types of organoclays [3,20].

The specific crystal structure and highly chemically reactive surface of montmorillonite, being the main component of bentonite, enables its properties to be changed by reacting with selected organic compounds, very often leading to the intercalated structures [21,22]. This gives the opportunity to develop a new precursor of the desired carbon structure as a result of the thermal destruction of the composite organic part without losing the bonding properties of the foundry binder. The correct selection of the organic modifier is a key step in the mineral modification process, which determines the level of ecological safety of the new inorganic-organic binder. Due to the promising physicochemical characteristics questioning the possibility of generating harmful compounds during high temperature pyrolysis, particular attention was paid to polymers from the group of acrylic compounds: poly(acrylic acid) (PAA) and its sodium salt (PAA/Na).

Poly(acrylic acid) (PAA) has a carboxyl group on every two carbon atoms of the main chain. The full dissociation of these functional groups provides the macromolecule with a high negative charge density [23,24,25]. The molar mass distribution of the acrylic polymers is broad, as they are industrially synthesized by radical polymerization of acrylic acid or sodium acrylic acid [26]. Together with poly(sodium acrylate), poly(acrylic acid) is one of the most commonly used water-soluble anionic polyelectrolytes, e.g., in the production of hydrogels, superabsorbents and ion exchange resins as well as the dispersing and binding agents [27,28,29]. Furthermore, due to low toxicity, they are used as a food additive [26]. Such properties as hydrophilicity, nontoxicity and binding capacity are valuable from the point of view of requirements of foundry binders [30]. Therefore, the selected polymers are promising materials in terms of the structure modification of the main bentonite component.

The following article attempts to identify the possible interactions connecting the inorganic and organic species of the obtained composites, i.e., montmorillonite in calcium bentonite modified with poly(acrylic acid) and its sodium salt. The analysis was performed with the use of basic instrumental research methods: FTIR spectroscopy and XRD analysis. The adsorption properties with the specific surface area of composites were determined performing BET nitrogen adsorption measurements.

## 2. Materials and Methods

### 2.1. Materials

Calcium bentonite (SN in the study) was supplied by ZGM Zębiec S.A. (Starachowice, Poland). It is characterized by the cation exchange capacity determined by the Cu(II)-TET adsorption method 65.3 meq 100 g^−1^ clay, MMT content 69.2% and swelling index 8 cm^3^ 2 g^−1^. The chemical composition of the unmodified SN bentonite is as follows: SiO_2_ 67.39%, Al_2_O_3_ 18.96%, MgO 4.58%, CaO 3.02%, Fe_2_O_3_ 2.73%, Na_2_O 1.28%, K_2_O 1.13%. Poly(acrylic acid) (PAA, average *M_w_* 1800 g mol^−1^) was purchased from Sigma-Aldrich (Poznan, Poland) and used without further purification. The high purity (≥99.0%) sodium carbonate, Na_2_CO_3_ (Sigma-Aldrich, Poznan, Poland), was used to form the following systems: SN/Na and PAA/Na intended for further preparation of the target composite materials. 

### 2.2. Preparation of Composite Materials

#### 2.2.1. Obtaining SN/PAA Composites

Organobentonite composites were prepared by modification of calcium bentonite with poly(acrylic acid). The previously prepared polymer solutions in concentration of 5, 15 and 25% by weight of bentonite were introduced into the pre-dispersed in the laboratory stirrer (300 rpm, 3 h) mineral suspensions of 5 g calcium bentonite per 100 mL of distilled water. Aqueous polymer solutions were prepared by dissolving the appropriate amount of PAA in 20 mL of distilled water. The mixtures were homogenized for 6 h in a laboratory stirrer at 300 rpm and then left to stand for a 1 week modification process. The stirring operation was repeated, and the resulting dispersions were centrifuged (8000 rpm, 12 min). After separation from the unreacted polymer, each organobentonite precipitate was dried to constant weight at 105 °C and then milled in an agate mortar. The first series of organobentonites were obtained: SN/5PAA, SN/15PAA, SN/25PAA.

#### 2.2.2. Obtaining SN/PAA/Na Composites

The second series of organobentonites, SN/5PAA/Na, SN/15PAA/Na and SN/25PAA/Na, was obtained by introducing aqueous solutions of poly(sodium acrylate) into the bentonite dispersion. The poly(sodium acrylate) solutions were prepared from the appropriate amount of PAA (5, 15, 25% by weight of bentonite) and the constant sodium carbonate (Na_2_CO_3_) content corresponding to the ion-exchange capacity (IEC) of the mineral. The further modification process was analogous to that described for calcium bentonite/poly(acrylic acid) SN/PAA materials. The procedure complemented the modification method related to the polymer addition into the mineral dispersion in the form of ion pairs with sodium ions Na^+^ [31]. According to the source literature, such action was supposed to have a beneficial effect on the polymer intercalation capabilities into the interlayer space of montmorillonite.

#### 2.2.3. Obtaining SN-Na/PAA Composites

The third, last series of organobentonites, namely SN-Na/5PAA, SN-Na/15PAA and SN-Na/25PAA, was prepared for comparison purposes. The modification procedure was analogous to that described for SN/PAA materials, whereby instead of calcium bentonite (SN), sodium-activated bentonite (SN-Na) was used. The activation process was carried out with the constant Na_2_CO_3_ content corresponding to the ion-exchange capacity (IEC) of the mineral, following the patent description of calcium bentonite activation [32].

### 2.3. Characterization Methods

The article is limited to the selected research techniques that ensured a sufficiently reliable assessment of the impact of three different types of organic modifications on the mineral crystal structure.

The chemical composition measurement of initial bentonite was carried out by X-ray fluorescence spectrometry (XRF) using Axios Max apparatus (Malvern Panalytical, Malvern, UK). The interaction between montmorillonite in bentonite and poly(acrylic acid) as well as its sodium salt solutions was assessed on the basis of the structural analysis results of Fourier-transform infrared spectroscopy (FTIR). The FTIR spectroscopy was performed in the mid-IR (MIR) region (4000–400 cm^−1^) using Digilab Excalibur FTS 3000MX spectrometer (Bio-Rad Laboratories GmbH, München, Germany). The standard transmission technique was used. The samples were measured in the form of KBr pellets with a sample/KBr ratio of 1/200. The spectra were recorded at 4 cm^−1^ resolution with 64 recorded sample scans. To perform the X-ray diffraction (XRD) measurements, an Empyrean diffractometer with a PIXcel3D detector (Malvern Panalytical, Malvern, UK) equipped with a copper anode CuK*α* source positioned in Bragg–Brentano geometry was used. The samples were prepared by the backloading method. XRD patterns were recorded over the angle range of 5–60°, with a step size of 0.017° (2*θ*) and CuK*α* radiation *λ* = 0.15406 nm.

The specific surface area of bentonite and obtained organobentonites, being one of the most important properties controlling their surface phenomena, was determined on the basis of the BET nitrogen adsorption method. The measurements were performed using the ASAP 2010 unit (Micromeritics Instrument Corporation, Norcross, GA, USA) with the N_2_ usage (99.999%, Air Liquide, Kraków, Poland). In order to remove all kinds of gases and impurities that may interfere with adsorption of N_2_ gas, the powdered samples were degassed at 120 °C for 24 h. Microstructural studies were performed with scanning electron microscopy (SEM) examination followed by electron dispersive spectroscopy (EDS) with the use of Phenom XL apparatus (Thermo Fisher Scientific, Waltham, MA, USA). Analyzed samples were sputtered with a very thin gold layer of ca. 10 nm thickness. A back-scattered electron detector and an acceleration voltage of 10 kV (for SEM images) or 15 kV (for EDS analysis) were applied.

## 3. Results and Discussion

### 3.1. Fourier Transform-Infrared Spectroscopy Analysis

**Figure 1:**
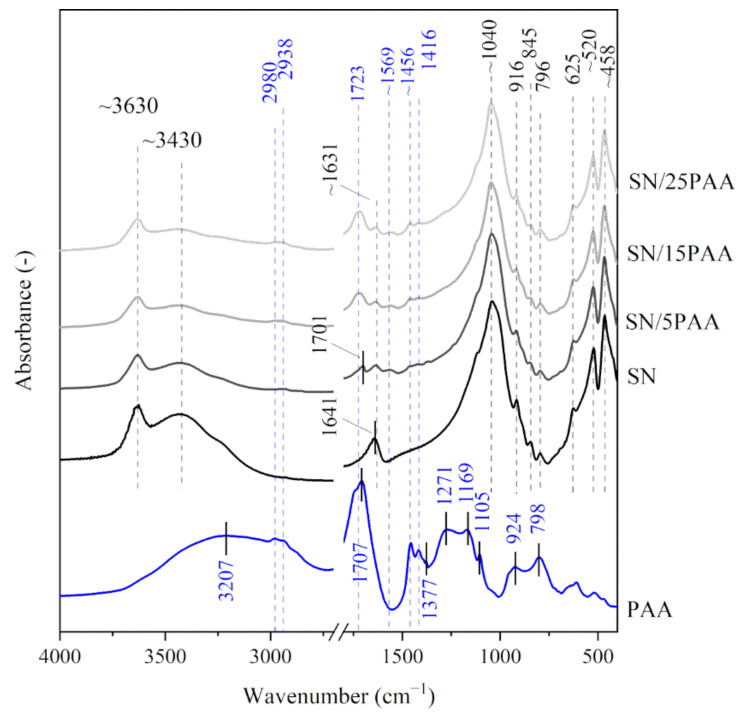
IR spectra of starting materials SN and PAA used in the preparation of composite materials with different polymer contents: SN/5PAA, SN/15PAA, SN/25PAA.

**Figure 2:**
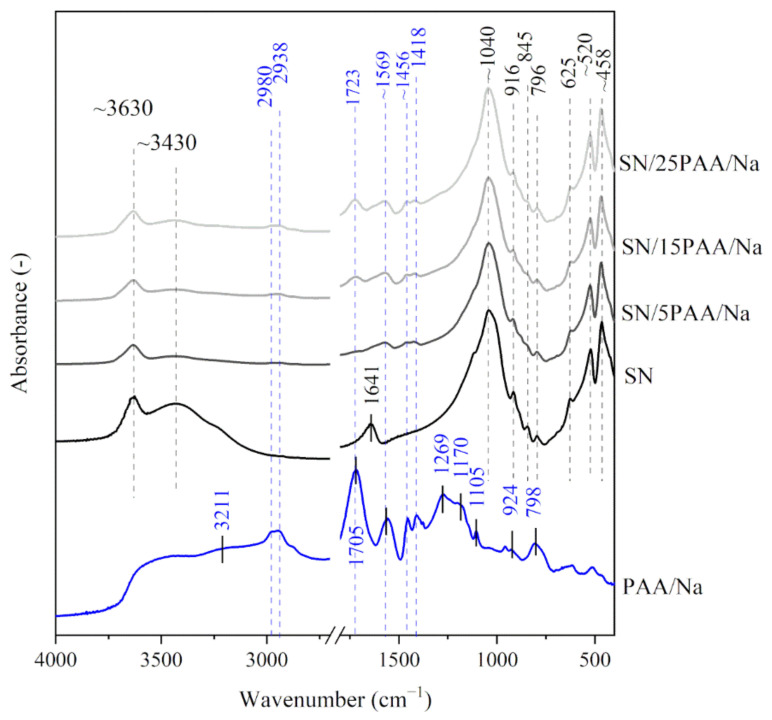
IR spectra of starting materials SN and PAA/Na used in the preparation of composite materials with different polymer contents: SN/5PAA/Na, SN/15PAA/Na, SN/25PAA/Na.

**Figure 3:**
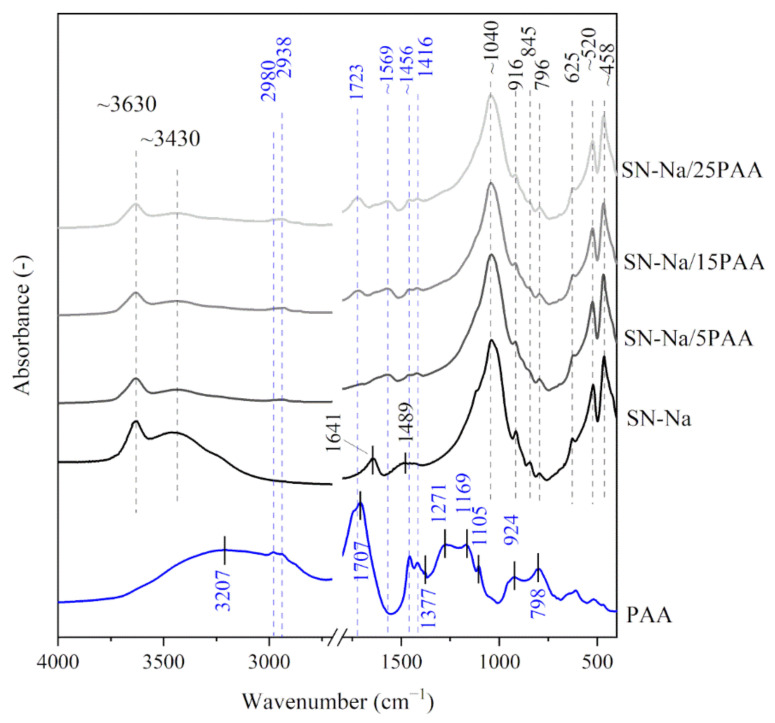
IR spectra of starting materials SN-Na and PAA used in the preparation of composite materials with different polymer contents: SN-Na/5PAA, SN-Na/15PAA, SN-Na/25PAA.

Figure 1 and Figure 2 show the IR spectra of calcium bentonite SN. The characteristic absorption band of the structural hydroxyl group –OH associated with Al^3+^ cations of octahedral montmorillonite (MMT) layers was observed at a wavenumber of 3631 cm^−1^. This maximum was also the first of three modes of water molecules vibrations (*ν*_1_, symmetrical stretching vibrations). The other modes of H_2_O vibrations corresponded to the absorption bands recorded at 3437 cm^−1^ (*ν*_2_, asymmetric stretching vibrations) and 1641 cm^−1^ (*ν*_3_, bending vibrations) [33]. The most intense band at 1043 cm^−1^ corresponded to Si–O asymmetric stretching vibrations in the tetrahedral forming the outer layers of aluminosilicate packages of MMT, while the band with a maximum at 796 cm^−1^ was associated with Si–O–Si symmetric stretching vibrations [34,35], indicating the presence of quartz in the samples (confirmed in the XRD test). The bands at 916 and 845 cm^−1^ were attributed to Al–Al–OH and Al–Mg–OH bending vibrations, respectively [36,37,38]. The three bands in the 600−400 cm^−1^ range represented –OH (625 cm^−1^) [39], Si–O–Al (520 cm^−1^) and Si–O–Si (458 cm^−1^) bending vibrations [36,40]. The IR spectra of sodium activated bentonite indicated the presence of analogous functional groups as recorded for SN, with the difference at the wavenumber of 1489 cm^−1^ proving the presence of CO_3_^2−^ carbonate ions from the sodium activation process (SN-Na, Figure 3) [41].

The IR spectra of poly(acrylic acid) displayed a band with a large half-width in the wavenumber range of 3500–2500 cm^−1^, which indicated the presence of intramolecular hydrogen bonds within the carboxyl group (3207 cm^−1^) (PAA, Figure 1) [42,43]. The bands with a maximum at 2980 and 2938 cm^−1^ were assigned with the C–H stretching vibrations [42,44,45,46]. The maximum of the highest intensity band at 1707 cm^−1^ was attributed to the stretching vibrations of the carbonyl group C=O, typical for PAA [30,47,48]. The absorption bands characteristic for the long carbon chains at 1456 and 1105 cm^−1^ were also recorded. The bands were assigned with the CH_2_ scissor vibrations near the carboxyl group and the C–C stretching vibrations of the main chain, respectively [25,41,42]. In the low wavenumber range (below 1000 cm^−1^), the presence of bands with the maximum at 924 and 798 cm^−1^ were associated with the occurrence of C–O stretching and CH_2_ wagging vibrations from the H–C group [40,44]. The spectrum course of the absorption bands of poly(acrylic acid) sodium salt was similar to the IR spectra of poly(acrylic acid). The main difference was noticeable at 1569 cm^−1^, which indicated the formation of carboxylate ions –COO^−^, typical for poly(acrylic acid) salts (PAA/Na, Figure 2) [39,45].

The interpretation of the organobentonites IR spectra was made by considering the different polymer contents within the three synthesis methods. Particular attention was paid to the analysis of the wavenumber range of 3800–3000 cm^−1^ and 1800–1400 cm^−1^, suggesting a possible polymer reaction with the mineral surface.

Figure 1 shows the absorption spectra for SN/PAA composites compared to the unmodified bentonite and poly(acrylic acid) The analysis of the obtained structural test results in the wavenumber range of 3800–3000 cm^−1^ indicated no hydrogen bond formation between the organic molecules and the montmorillonite crystal structure. However, the partial desorption of the interlayer water and the water adsorbed on the mineral surface was recorded. It was suggested by the decrease in band intensity with the maximum at ~3430 cm^−1^ and the change in shape and the shift towards the lower wavenumber of the maximum band corresponding to the bending OH vibrations at 1640 cm^−1^, respectively.

The increasing band intensity at about 1700 cm^−1^ on the IR spectra of SN/5PAA, SN/15PAA and SN/25PAA composites, which corresponded to the vibrations of the characteristic for PAA C=O groups, indicating an increasing polymer concentration in the organobentonites (Figure 1). The band was shifted towards higher wavenumber values (1723 cm^−1^) compared to its position on the absorption spectrum of pure polymer (1707 cm^−1^), which proved the changes taking place within the carbonyl group. The new band with a maximum at 1569 cm^−1^ was attributed to the asymmetric stretching vibrations of the carboxylate anion –COO^−^, indicating the low degree of ionization of the carboxyl groups. Its intensity decreased in the direction of SN/5PAA → SN/25PAA, which resulted directly from the increasing concentration of the polymer in the composites, and thus their decreasing pH from about 3.0 to about 2.5.

The hydroxyl-stretch region for SN/PAA/Na composites (3800–3000 cm^−1^), shown in Figure 2, revealed no significant changes in the position and intensity of the identified bands in comparison with the IR spectra of unmodified clay. Nevertheless, similarly to the SN/PAA materials, a high degree of montmorillonite interlayer water desorption occurred.

The IR spectra of the SN/PAA/Na organobentonites showed the presence of the –COO^−^ band at 1569 cm^−1^, which was already clearly visible at 5% PAA/Na content. The band intensity increased with the increase of the polymer percentage. This may suggest a higher ionization degree of the C=O functional groups in SN/PAA/Na materials compared to the SN/PAA composites with an appropriate corresponding content of the organic modifier.

The analysis of the shape and position of the absorption bands of sodium activated bentonite modified with the poly(acrylic acid), i.e., SN-Na/5PAA–SN-Na/25PAA composites in the range of 3800–3000 cm^−1^, showed a similar desorption of the interlayer water molecules as recorded for the other two organobentonite groups (Figure 3). The courses of the IR spectra in the wavenumber range of 1800–1400 cm^−1^ were comparable to those obtained for SN/PAA/Na materials, which suggests no influence of the modification method on the interaction process of poly(acrylic acid) and the mineral structure (Figure 2).

**Figure 4:**
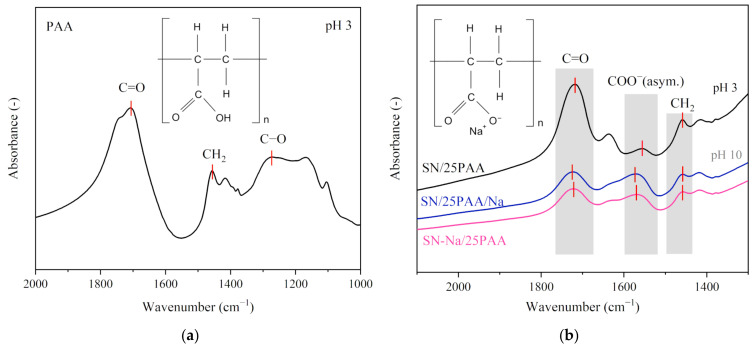
(**a**) IR spectra of poly(acrylic acid); (**b**) IR spectra of SN/25PAA, SN/25PAA/Na, SN-Na/25PAA composites in the wavenumber range corresponding to the presence of the polymer carboxyl group and carboxylate ion.

Figure 4 displays the selected wavenumber range of the absorption spectra recorded for the composites containing 25 wt.%. A compilation of the IR spectra revealed the intensity disproportion of the bands assigned to the C=O and –COO^−^ vibrations in the SN/25PAA materials in comparison to the SN/25PAA/Na and SN-Na/25PAA composites, which indicated a lower ionization degree of the polymer molecules.

A high ionization degree of the C=O groups observed in the SN/PAA/Na IR spectra resulted from the composite’s high pH, reaching a value above 8.0. Under such reaction conditions, the polymer became an anionic polyelectrolyte, which, due to the similarity of the electric charges, could not interact with the aluminosilicate surface at the molecular level [25,47]. However, the inspiration for such a mineral modification was the theoretical possibility of introducing an anionic organic compound into the interlayer space of montmorillonite in the form of an ion pair with Na^+^ cations, which was supposed to favor the polymer intercalation process with the simultaneous ion exchange of Ca^2+^ interlayer cations [31].

In the range of low wavenumbers (below 1450 cm^−1^), no changes in the positions of absorption bands of organobentonites in relation to those recorded for the unmodified bentonite spectrum were observed (Figure 1, Figure 2 and Figure 3). This is a direct consequence of maintaining the initial crystal structure of the mineral [49].

### 3.2. X-ray Diffraction Analysis

**Figure 5:**
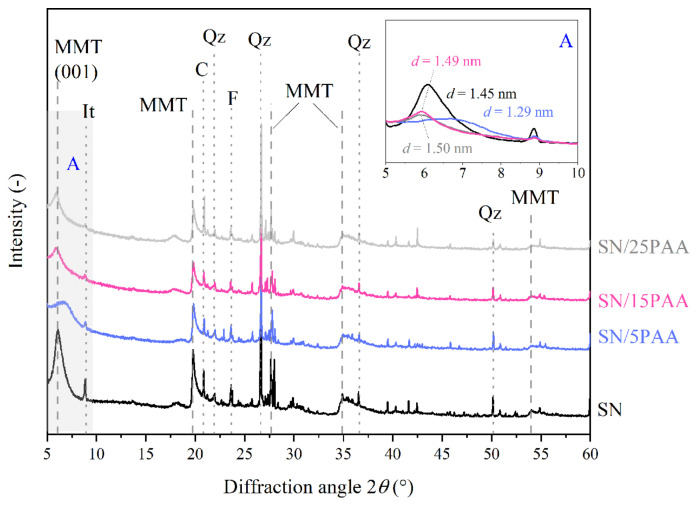
X-ray diffraction patterns of unmodified bentonite SN and modified with poly(acrylic acid) bentonite SN/PAA with distinction into the polymer content (MMT—montmorillonite, It—illite, C—calcite, Qz—quartz, F—feldspar). The A area is a magnification of the XRD pattern in the angle range of 5–10°.

**Figure 6:**
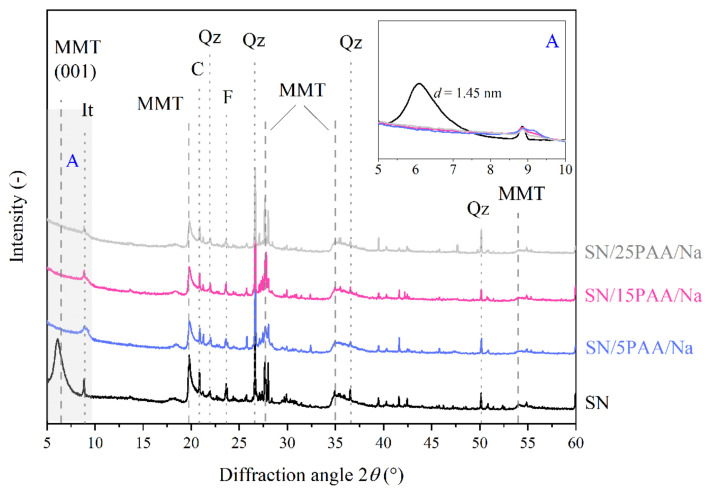
X-ray diffraction patterns of unmodified bentonite SN and modified with poly(acrylic acid) sodium salt bentonite SN/PAA/Na with distinction into the polymer content (MMT—montmorillonite, It—illite, C—calcite, Qz—quartz, F—feldspar). The A area is a magnification of the XRD pattern in the angle range of 5–10°.

**Figure 7:**
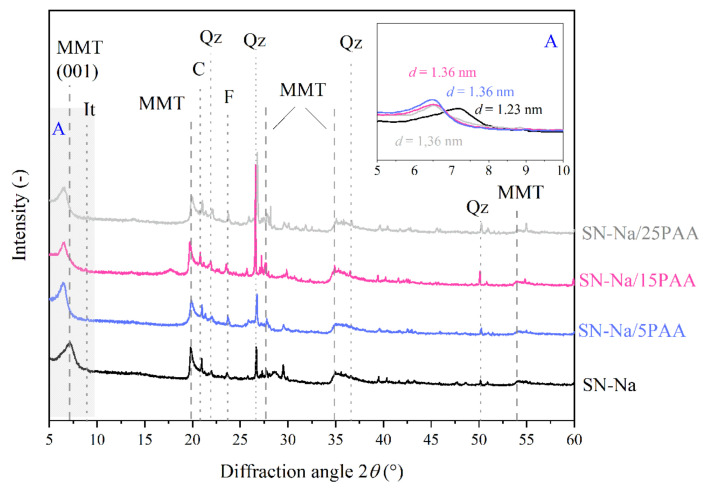
X-ray diffraction patterns of sodium bentonite SN-Na and modified with poly(acrylic acid) bentonite SN-Na/PAA with distinction into the polymer content (MMT—montmorillonite, It—illite, C—calcite, Qz—quartz, F—feldspar). The A area is a magnification of the XRD pattern in the angle range of 5–10°.

The XRD pattern of the unmodified bentonite SN reveals the presence of the diffraction peak at 2*θ* = 6.1°, which is directly associated with the basal spacing between montmorillonite packages, *d*_001_, of 1.45 nm (Figure 5 and Figure 6). The mineral interlayer distance in the sodium activated bentonite SN-Na reached a value of *d*_001_ = 1.23 nm at the detector angle 2*θ* = 7.2° (Figure 7). The position change analysis of the diffraction peak, corresponding to the aluminosilicate interlamellar spacing, was crucial in determining the influence of the organic modification on the mineral structural properties.

The modification of calcium bentonite with a poly(acrylic acid) led to an increase in the interlayer distance of montmorillonite to the *d*_001_ value of about 1.50 nm, most likely creating a monolayer (Figure 5). However, the PAA intercalation was possible with its appropriate weight ratio to the mineral, as only the desorption of an interlayer water molecules for the SN/5PAA material was recorded. The low intensity of the diffraction peak attributed to the interlayer spaces of a regular stacking of the silicate layers along the (001) direction for the SN/15PAA and SN/25PAA organobentonites suggested the low plane repeatability. It might be the result of the repulsion of like negative charges at mineral packages and polymer chains, some of which, due to the reaction with a small amount of Na^+^ cations placed at the edge of the interlayer space, obtained the sodium form incapable of intercalation. Such a phenomenon is suggested by the previously analyzed results of the FTIR structural studies, showing the presence of an absorption band (1569 cm^−1^) typical for the sodium salt of poly(acrylic acid) in the SN/PAA composites (Figure 1).

The phase analysis of the SN/PAA/Na X-ray diffractograms showed no peak in the range of angle 2*θ*: 5–10°, typical for the MMT layered structure (Figure 6). This suggests a destructive effect of the poly(acrylic acid) sodium salt on the crystal structure of the modified aluminosilicate, thus limiting its most important properties for the foundry industry, e.g., binding capacity.

XRD analysis confirmed the formation of an intercalated structures in the form of SN-Na/PAA organobentonites already at 5% of the polymer content (Figure 7). The interlayer platelet spacing of montmorillonite increased from 1.22 nm for the SN-Na to 1.36 nm for the SN-Na/PAA materials, regardless of the organic modifier concentration.

Other peaks corresponding to the montmorillonite phase in bentonite were observed at 2*θ* of 20.0°, 27.2° (101), 34.9° (102) and 54.2° (110). The reflexes at 22.0°, 26.8°, 36.7° and 50.1° 2θ angles were associated with quartz (*Qz*). Such impurities as feldspar (*F*, 2*θ* = 23.6°) and calcite (*C*, 2*θ* = 20.9°) were also present in the bentonites and all types of organobentonites [50,51,52].

### 3.3. BET Surface Area Analysis

It is considered that the BET nitrogen adsorption method provides information about the mineral external surface area only (SA_BET_ in this study), although many scientists are critical of this statement. Despite the good reproducibility of the BET technique, the key issue remains the actual adsorption sites of N_2_ molecules [53,54]. One of the most important factors determining the SA_BET_ value are the basic planes forming the layered structure of the minerals and their micro and mesoporosity [55]. The microporosity corresponds to the edges of the packages and results from the imperfections in the T–O–T layer stacking [56]. The mesopores are formed by the irregular stacking of the elementary mineral particles and its aggregates [55]. Initially, the dependence of montmorillonite microporosity on the *d*_001_ value related to the type of exchangeable ions was assumed. Due to this phenomenon, the difference between the SA_BET_ value of SN bentonite (36.09 m^2^·g^−1^) and activated SN-Na bentonite (25.12 m^2^·g^−1^) could have been explained. However, it turns out that it was not the basal spacing of montmorillonite, but the above-mentioned mutual arrangement of the T–O–T layers, as well as the layer charge density (LCD) that had a key impact on the mineral BET surface area. Thus, the higher LCD value of bentonite SN, related directly to the presence of natural divalent Ca^2+^ interlayer cations, created conditions favoring the adsorption of N_2_ molecules [53].

Table 1 summarizes the results of the surface area (SA_BET_) measurements conducted for calcium, sodium and organic modified bentonite clays.

Regardless of the organic modification method, the BET surface area of the organobentonites was smaller compared to the starting materials, i.e., SN and SN-Na bentonites. The SA_BET_ average of SN/5PAA, SN/5PAA/Na and SN-Na/5PAA composites could be considered equal to 8.0 m^2^·g^−1^. This confirms the well-established process of the polyelectrolyte chains adsorption onto the mineral surface [57]. The observed further decrease in the SA_BET_ value of organobentonites along with the increase in the polymer content were attributed to the formation of coiled shape poly(acrylic acid) molecules coating mineral particles [57,58]. The formation of tightly folded polymer coils, held together by the numerous cohesive and attractive both intra- and intermolecular forces, was intensified in the saline environment [26]. The adsorption mechanism may be either the electrostatic nature between the negative charges of the polymer and the positively charged edges of the mineral particles, or it may be the result of an ion exchange between the OH^−^ ions of the montmorillonite particle faces and the anionic part of the PAA chains [57].

### 3.4. Scanning Electron Microscopy and X-ray Microanalysis

The SEM/EDS analysis included organobentonites with a 25 wt.% of the organic part due to their expected representativeness in terms of the general polymer behavior at the mineral surface.

**Figure 8:**
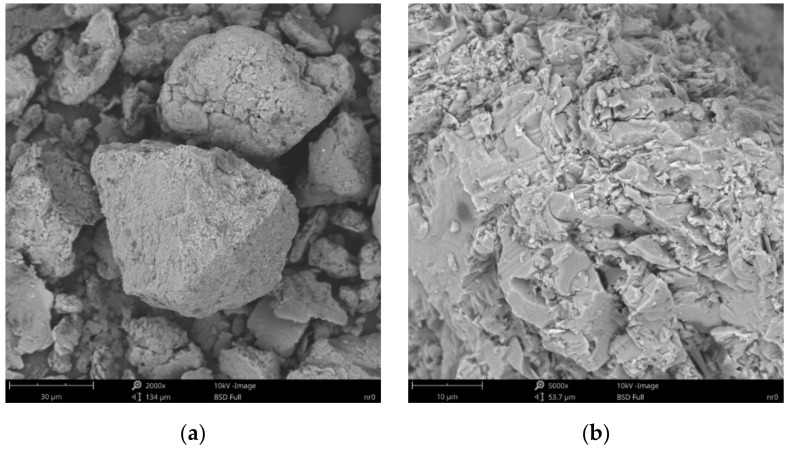
SEM images of particle surfaces for (**a**) SN, magnification: 2000×, scale bar: 30 μm; (**b**) SN, magnification: 5000×, scale bar: 10 μm.

**Figure 9:**
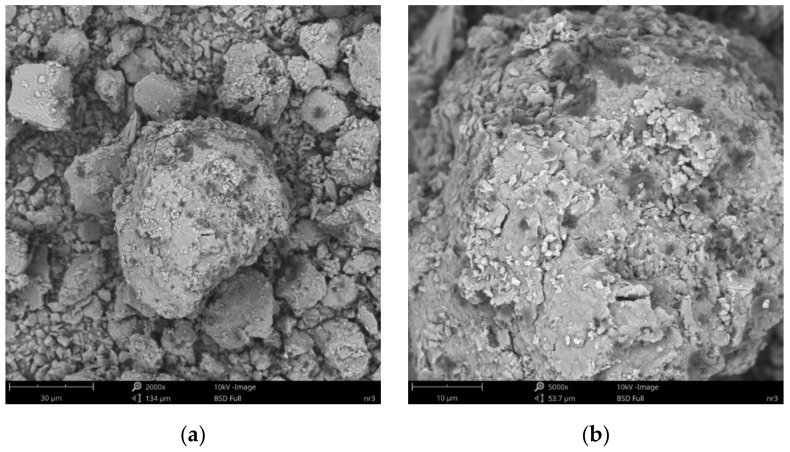
SEM images of particle surfaces for (**a**) SN/25PAA, magnification: 2000×, scale bar: 30 μm; (**b**) SN/25PAA, magnification: 5000×, scale bar: 10 μm.

**Figure 10:**
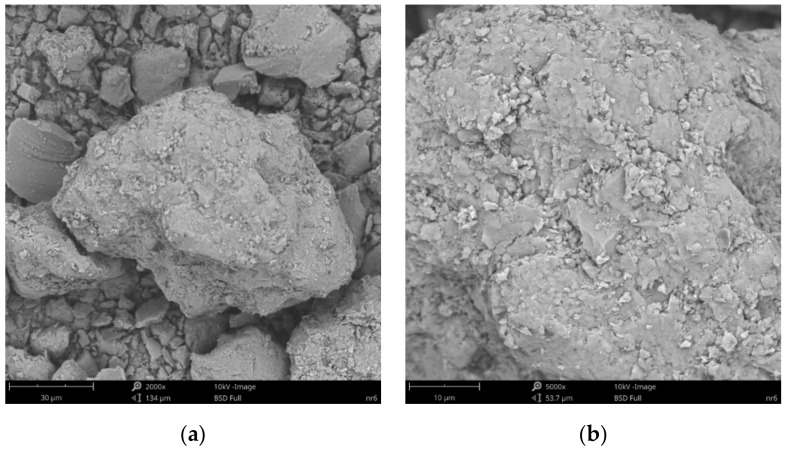
SEM images of particle surfaces for (**a**) SN/25PAA/Na, magnification: 2000×, scale bar: 30 μm; (**b**) SN/25PAA/Na, magnification: 5000×, scale bar: 10 μm.

**Figure 11:**
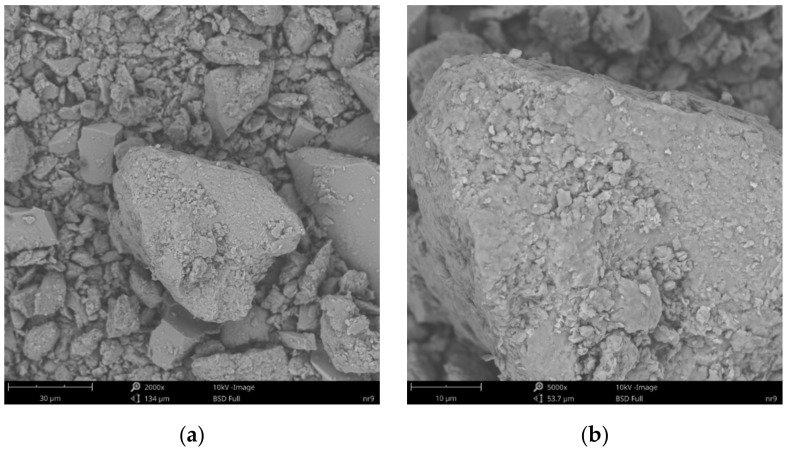
SEM images of particle surfaces for (**a**) SN-Na/25PAA, magnification: 2000×, scale bar: 30 μm; (**b**) SN-Na/25PAA, magnification: 5000×, scale bar: 10 μm.

SEM images of the microscopic surfaces of initial SN mineral and SN/25PAA, SN/25PAA/Na and SN-Na/25PAA composites prepared by the organic modification are presented in Figure 8, Figure 9, Figure 10 and Figure 11, respectively.

Figure 8b reveals the lamellar structure of unmodified bentonite with visible edges, which was the result of the discontinuity of its layered structure. Phase separations were observed as heterogeneous surface morphology. Based on the visual analysis of SEM images obtained for the SN/25PAA composite, the presence of adsorbed and well-dispersed polymer molecules (bright dots) on the surface of the mineral particle was confirmed (Figure 9a). The coiled shape poly(acrylic acid) molecules were adsorbed, most probably via electrostatic forces, covering mineral agglomerates. This phenomenon was less visible in the case of the other two types of organobentonites, i.e., SN/25PAA/Na and SN-Na/25PAA (Figure 10a and Figure 11a) Nevertheless, all composite particles were characterized by small irregularities and bumps, causing an increased degree of the surface roughness, which indicates the polymer influence on the crystal structure of the mineral (Figure 9b, Figure 10b and Figure 11b).

**Figure 12:**
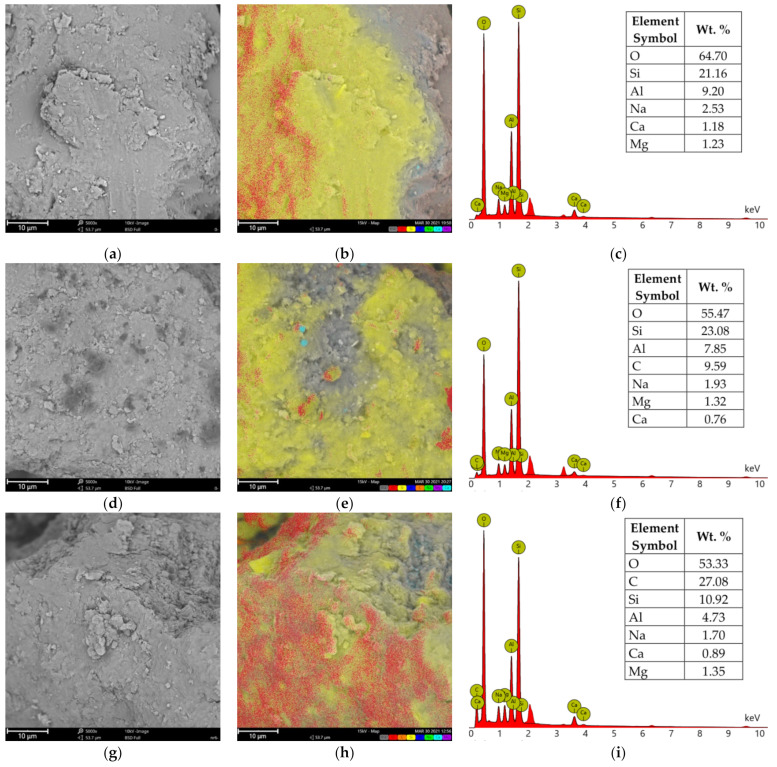

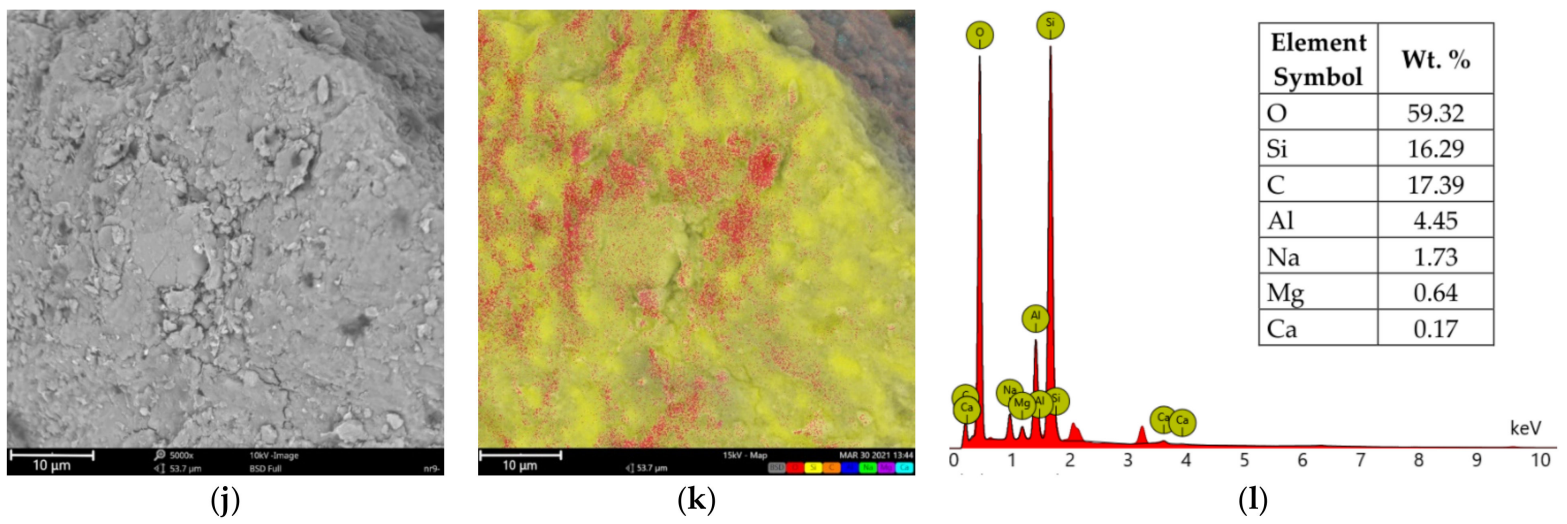
SEM images of (**a**) SN, (**d**) SN/25PAA, (**g**) SN/25PAA/Na, (**j**) SN-Na/25PAA materials, respectively; elemental distribution of (**b**) SN, (**e**) SN/25PAA, (**h**) SN/25PAA/Na, (**k**) SN-Na/25PAA materials, respectively (element color: O—red, Si—yellow, Al—dark blue, C—orange, Na—green, Mg—purple, Ca—light blue); EDS analysis of (**c**) SN, (**f**) SN/25PAA, (**i**) SN/25PAA/Na, (**l**) SN-Na/25PAA materials, respectively. The analysis was performed at a magnification of 5000×.

Chemical composition analysis (EDS) of calcium bentonite revealed the presence of silicon (Si), which in the form of silicate (SiO_2_) is the main component of this form of clay, followed by aluminum in an alumina form (Al_2_O_3_) (Figure 12a–c). The other minor elements of the alkali groups, including calcium (Ca), sodium (Na) and magnesium (Mg) may also be found as constitutional elements of SN. The same elemental composition was determined at the surfaces of SN/25PAA, SN/25PAA/Na and SN-Na/25PAA composites (Figure 12d–f,g–i,j–l, respectively). Additionally, carbon (C) presence on the modified bentonite samples was confirmed, which indicates the surface adsorption of polymer molecules. It may be noted that in the case of SN/25PAA and SN-Na/25PAA, a smaller share of carbon was found on the mineral surface compared to the SN/25PAA/Na material. Considering the same polymer content in the composites and the results of XRD studies, it may suggest the partial intercalation of PAA chains between montmorillonite layers in SN/25PAA and SN-Na/25PAA organobentonites.

**Figure 13:**
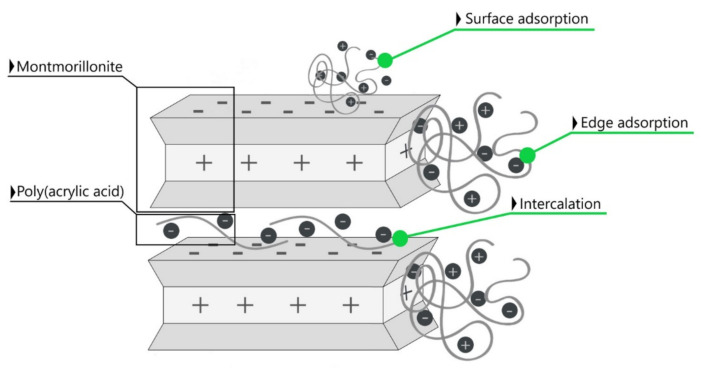
Scheme of some of the possible interactions of montmorillonite in calcium- and sodium-activated bentonite with the chains of poly(acrylic acid): surface and edge adsorption combined with the intercalation.

The BET results (specific surface area data) in combination with the FTIR, XRD measurements (structural analysis) and SEM images (morphology analysis) indicated the physical bonding of poly(acrylic acid) to the outer surface formed by the tetrahedral layers as well as the edges of all layers of the calcium and sodium-activated montmorillonite. As a result of numerous isomorphic substitutions in the mineral structural sheets, its particles had negative charges at the basal surfaces and positive charges at the edges, which provides conditions for a specific particle interaction like edge-to-face, edge-to-edge and face-to-face [59]. Therefore, the interaction between clay particles and polymer molecules is based on the charge attraction [60]. The adsorption of polymer chains was accompanied by its intercalation into the mineral structure in the form of a monolayer, causing an increase in the interlayer space, which is schematically presented in Figure 13.

## 4. Conclusions

The article aims to verify the possibility of obtaining a material capable of binding the mineral matrix grains in molding sand technology, which could also act as a lustrous carbon carrier. This material, due to the form of organoclay, widely used in other industries, compared to the available substitutes for commonly used carbon additives based on multi-component systems, but still containing coal dust, may be a new approach to solving the problem of the harmfulness of bentonite-bonded sands.

The paper presents a combined experimental study of two-component composites based on calcium bentonite modified with poly(acrylic acid) and poly(acrylic acid) sodium salt. To determine the effect of the interlayer ion types on the change in the mineral structure, an organic modification of sodium-activated bentonite using poly(acrylic acid) was also carried out. The composites contained up to 25 wt.% of polymer.

The structural analysis revealed no chemical bonding of the polymer chains to the montmorillonite surface, regardless of the type of organic modification. The interactions between the components of the analyzed organobentonites are mainly of a physical nature. The increase in the interlayer distance between the silicate layers of organobentonites indicates the intercalation of a polymer monolayer, whereby the *d*_001_ parameter was not particularly dependent on the content of the organic modifier. The intercalation of poly(acrylic acid) into the interlayer space of montmorillonite is advantageous from the development perspective of a new foundry binder, which could also act as a carrier of the desired carbon structure. The modification of calcium bentonite with the poly(acrylic acid) sodium salt resulted in the destruction of the mineral′s layered structure, causing the loss of a valuable binding property for foundry binders. The BET measurements showed a reduction in the specific surface area of all organobentonites, indicating the coating of mineral particles with polymer molecules. The surface adsorption was also confirmed by SEM/EDS analysis. Considering the anionic nature of poly(acrylic acid) chains in the reaction environment, their electrostatic interaction with the positively charged edges of the mineral sheets seems to be the most likely. Although surface adsorption of poly(acrylic acid) molecules can adversely affect, for example, the swelling capacity of the bentonite, the good binding properties of the polymer itself can overcome the potential limitations. However, it may turn out that the quantity of the desired carbon structure formed while pouring the mold with liquid metal or the thermal properties of organobentonites will not be sufficient to act as binders in the foundry technology. The qualitative modification of the material composition should then be considered.

The obtained experimental results will be considered in the further stages of research, in which the modified bentonites will be used as a binder in synthetic molding sands. Selected physicochemical properties of molding sands with a new binder will be studied.

## Figures and Tables

**Table 1:** Surface area (SA_BET_) of SN and SN-Na bentonites and their organic modifications.

Composite	SA_BET_ (m^2^·g^−1^)	Composite	SA_BET_ (m^2^·g^−1^)	Composite	SA_BET_ (m^2^·g^−1^)
SN/5PAA	7.71	SN/5PAA/Na	8.78	SN-Na/5PAA	7.35
SN/15PAA	5.06	SN/15PAA/Na	6.37	SN-Na/15PAA	4.49
SN/25PAA	2.71	SN/25PAA/Na	2.45	SN-Na/25PAA	2.73

## Data Availability

The data are contained within the article and/or available on request from the corresponding author.
